# Comparative dosimetry of GammaMed Plus high-dose rate ^192^Ir brachytherapy source

**DOI:** 10.4103/0971-6203.66761

**Published:** 2010

**Authors:** N. P. Patel, B. Majumdar, V. Vijayan

**Affiliations:** 1 Department of Physics, Govt. College of Science, Raipur; India; 2 Department of Medical Physics, A. H. Regional Cancer Center, Cuttack; India; 3 Institute of Physics, Bhubaneswar, India

**Keywords:** 0.1-cc chamber, AAPM TG-43, dosimetry, EGSnrc simulation code, high dose rate

## Abstract

The comparative dosimetry of GammaMed (GM) Plus high-dose rate brachytherapy source was performed by an experiment using 0.1-cc thimble ionization chamber and simulation-based study using EGSnrc code. In-water dose measurements were performed with 0.1-cc chamber to derive the radial dose function (r = 0.8 to 20.0 cm) and anisotropy function (r = 5.0 cm with polar angle from 10° to 170°). The nonuniformity correction factor for 0.1-cc chamber was applied for in-water measurements at shorter distances from the source. The EGSnrc code was used to derive the dose rate constant (Λ), radial dose function g_L_(r) and anisotropy function F(r, θ) of GM Plus source. The dosimetric data derived using EGSnrc code in our study were in very good agreement relative to published data for GM Plus source. The radial dose function up to 12 cm derived from measured dose using 0.1-cc chamber was in agreement within ±3% of data derived by the simulation study.

## Introduction

The GammaMed (GM) Plus is one of the high-dose rate ^192^Ir brachytherapy sources commonly used for the management of most of the malignancies. The acquisition of dosimetric data for the brachytherapy sources is essential for the treatment planning purposes. The treatment planning system performs the optimizations on applicator or image-based dose distribution to achieve the desired dose levels at specified points of clinical interest.[1] The American Association of Physicists in Medicine (AAPM) Radiation Therapy Committee Task Group (TG) No. 43 has recommended a set of dosimetric data for the high-dose rate brachytherapy sources.[2] Several authors have used various dosimetry systems to generate dosimetric data for ^192^Ir high-dose rate (HDR) brachytherapy sources.[3–16] These data vary with the type of the source as these are much dependent on the size of the active core of the source, isotope distribution, encapsulation material and its geometry. Even the comparative dosimetry of one type of source with two different dosimetry systems has shown deviations in data of about 5.0%.[4,5]

Monte Carlo calculation is a much reliable and preferable dosimetry system; it is widely used in deriving the dosimetric data for brachytherapy sources. The literatures related to the Monte Carlo (MC) simulation dosimetry of GammaMed Plus and other high-dose rate brachytherapy sources have been cited.[4–11] Ballester *et al*. have used GEANT code for the complete dosimetry of the GammaMed Plus HDR source.[9] Taylor and Rogers have used the EGSnrc user-code BrachyDose to derive the TG-43 dosimetry parameters for the GammaMed Plus HDR source.[10] Toye *et al*. have made a comparative dosimetry of Nucletron ‘classic’ ^192^Ir HDR source by MOSFET measurements in a water phantom and using the EGSnrc code.[11]

The ionization chambers have been used for different purposes in dosimetry of the high-dose rate ^192^Ir brachytherapy sources.[12–16] The major demerits of the ionization chamber are the inconvenience in use and the nonuniformity correction factor due to high dose gradient near the brachytherapy source. Meisberger *et al*. and Meli *et al*. have used the 0.1-cc chamber to derive the tissue attenuation factor from water-to-air dose measurement.[12,13] Meli *et al*. have concluded that the dose rate function derived from tissue attenuation factor is more accurate than the depth-dose measurement.[13] Venselaar *et al*. have used 0.6-cc chamber for dose measurement at larger distances from the source where the effect of nonuniformity correction factor of the chamber is minimum.[14] Nehru *et al*. have performed experimental study to derive radial dose function where measured dose at different distances were normalized relative to the dose at 5.0 cm from the source.[16] In the present study, we have used 0.1-cc ionization chamber for in-water dose measurement of GammaMed Plus ^192^Ir brachytherapy source. The positive aspect of our study is the dose determination by applying the nonuniformity correction factor for 0.1-cc ionization chamber at shorter distances from the source. The purpose is to evaluate the feasibility of its use by comparing the dosimetric parameters relative to derived data from simulation study. In the simulation study, we have used DOSRZnrc, and FLURZnrc (user codes of EGSnrc) for the dosimetry of the GM Plus source to determine the dose rate constant, radial dose function and anisotropy function. We have restricted our study to derive only some selected data to compare with the published data.

## Materials and Methods

### AAPM TG-43 dose calculation formalism

The dosimetric quantities calculated around the GammaMed Plus HDR brachytherapy source are dose rate constant (Λ), radial dose function *g_L_*(*r*) and anisotropy function *F*(*r*, θ) as defined by AAPM TG-43.[2]

### Monte Carlo simulation

The FLURZnrc and DOSRZnrc (user codes of EGSnrc, version 4) Monte Carlo simulation codes were used for calculation of air kerma strength and absorbed dose to water, respectively.[17–20] The EGSnrc code is a modified version of EGS4 code, where most of the algorithm and physics theory has been changed to enhance the accuracy of dose calculation for electron-scattering and low-energy photons.[17,21,22]

### GammaMed Plus source

The details of construction design and material composition of GammaMed Plus source used in our study are taken from the publication by Ballester *et al*.[9] The material composition of source active volume consists of 70% Ir and 30% Pt with an effective density of 21.76 g/cm^3^. The length and diameter of an active core of source were 0.35 and 0.06 cm, respectively. The lateral and top sides of the active core were hollow. The inner and outer diameters of stainless steel encapsulation were 0.07 and 0.09 cm, respectively. The encapsulation material was stainless steel (AISI 316 L) of density 8.06 g/cm^3^. The density of stainless steel cable of full length is taken as 5.6 g/cm^3^. The simulation of geometry in DOSRZnrc code is based on the RZ (radius-plane) coordinate, which has a limitation in the simulation of actual geometry of the tip of the source. The tip of the source with conical shape was simulated as three cylindrical slices of thicknesses 0.005, 0.005, 0.006 cm with corresponding radii of 0.012, 0.03 and 0.035 cm, respectively.

### Air kerma strength

The GM Plus source was simulated at the center of the cylindrical volume (10 m diameter and 10 m height) consisting of air with density of 0.12 g/cm^3^. The scoring cells for photon fluence were created in cross-sectional ring geometry at various distances from 1 to 100 cm along the transverse axis from the center of the source. The dimensions (ΔZ, ΔR=ΔY) of the scoring cells vary from 0.1 × 0.04 cm^2^ at 1.0 cm to 0.1 × 0.1 cm^2^ at 100 cm. First the geometry simulation was carried out in DOSRZnrc code; after that, the photon fluence simulation was performed in FLURZnrc user code. The bare ^192^Ir photon spectrum (Ir192_Bare_1993 photon spectrum) taken from Duchemin and Coursol was simulated.[23] The number of photon histories simulated was 10^9^. The size of energy bins used for the simulation varies from 5 to 40 KeV depending upon the photon fluence in the spectrum. The global transport cut-off energy for electron (AE) and photon (AP) was taken as 2.0 and 0.001 MeV, respectively. The air kerma strength was calculated as per the methodology described by Borg *et al*,[24] and using X-ray mass energy absorption coefficient for dry air taken from the publication by Hubbell and Seltzer.[25] The air kerma measurements at various distances between 10 and 100 cm were corrected for attenuation and scattering of primary photon by air[26]; then the corrected values were used to calculate air kerma strength of the source as per the method described by Williamson *et al*.[6]

### Dose calculation in water medium

The GammaMed Plus source was geometrically simulated at the center of the cylinder of dimensions 60 cm height and 60 cm diameter, consisting of liquid water. The anisotropy function *F*(*r*, θ) was generated for 1.0, 2.0, 3.0, 5.0 and 10.0 cm with polar angles varying from 0° to 175° and radial dose function *g_L_*(*r*) was generated for various distances between 0.08 and 20 cm. The polar coordinates (*r*, θ) of dose calculation points anisotropy functions were converted into Cartesian coordinates (*X, Y*). The center of the active core of the source was taken as origin. The dose-scoring cells are defined at Cartesian coordinates in cylindrical cross-sectional rings. The size (ΔZ, ΔR=ΔY) of the scoring regions varies with the distance from the source. The size range varies from 0.01 × 0.01 cm^2^ at 1.0 cm to 0.01 × 0.4 cm^2^ at 5.0 cm for the calculation of anisotropy function. For radial dose function, the size of cells varies from 0.02 × 0.004 cm^2^ at 0.8 cm to 0.02 × 0.08 cm^2^ at 20.0 cm. The total number of scoring cells useful for dose calculation was 98. The calculations were performed in three different batches due to limitations in the user code and to avoid overlapping of planes and cylinder of two different scoring cells. The global transport cut-off energy for electron (AE) and photon (AP) was taken as 0.01 and 0.001 MeV, respectively. The number of photon histories simulated in each batch was 10^9^. The statistical accuracy achieved in dose calculation in dose-scoring zones along transverse axis varied from 0.7% at 1.0 cm to 1.4% at 20.0 cm from the source. The statistical accuracy in dose-scoring zones offside the transverse axis increases with increase in angle. The dose was calculated for scoring zones within polar angle range of 15° to 165° irrespective of radial distances, with statistical accuracy of within 3%.

### In-water dose measurement using 0.1-cc chamber

**Figure 1:**
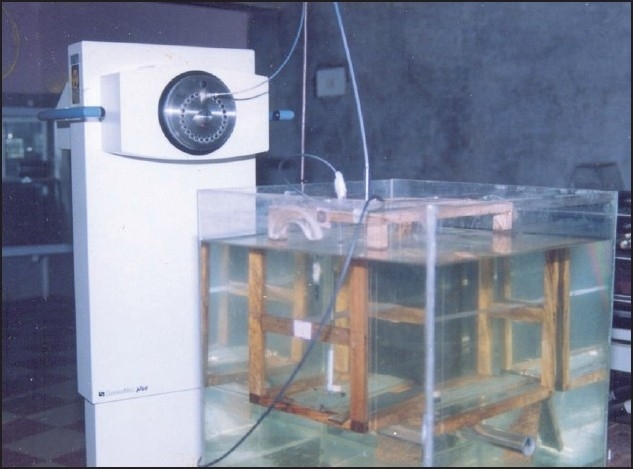
GammaMed Plus high-dose rate brachytherapy unit and measurement jig placed inside the water phantom

The PTW semiflex 0.1-cc ionization chamber was used in the experimental study, which has dimensions of sensitive volume of 0.325 cm diameter and 1.12 cm length. The measurement jig was made of low-scattering materials, viz., wood and Perspex. The detailed explanation about measurement jig and experimental procedure has been revealed in the publication.[27] The dimensions of water phantom were about 50 × 50 × 45 cm^3^, and the walls were made of acrylic. The jig was designed in such a way that it could be easily placed from air into water phantom (or vice versa) without any displacement of the chamber and the source applicator. The measurement setup showing the jig placed inside the water phantom is shown in Figure 1. The jig was usually placed inside the water phantom after setting the source applicator at measuring distance. The source stopping time at reference dwell position was set for more than the measuring period. The integrated charge was measured for a specified period. The transit time correction was not required as the source was stable during the charge collection.

### Tissue attenuation factor

The tissue attenuation factor is defined as the ratio of dose in water to that in air measured for constant source-to-chamber distance and in similar conditions. This factor was commonly used for brachytherapy treatment planning before the dosimetric data were recommended by AAPM TG-43. The tissue attenuation factors were measured along the transverse axis for the source-to-chamber distances between 0.8 and 20 cm. In our measurement, first the source applicator was placed at distance r from the chamber, and the measurement was performed for a specified period in air. After that, the jig was placed inside the water medium without displacing the chamber and source applicator, and the measurement was performed for the same period. The measured quantities in air were corrected for room scatter.[28] The tissue attenuation factors were then derived using the following formula:

.......(1) $$\begin{array}{l} \mathrm{Tissue}\;\mathrm{attenuation}\;\mathrm{factor}\;\alpha (r)\;=\frac{\mathrm{Dose}\;\mathrm{in}\;\mathrm{water}}{\mathrm{Dose}\;\mathrm{in}\;\mathrm{air}} \end{array}$$

### Radial dose function

We used two different methods to derive the radial dose function in our experimental study, which are described below:

#### Using tissue attenuation factor

In this method, we have used the formula described by Meli *et al*. to derive the radial dose function from tissue attenuation factor.[13] The formula is given by

.......(2), $$g_{L}\;\mathrm{(r)}\;=\;\frac{f\;(r)\;\alpha \;(r)}{f\;(1)\;\alpha (r)}$$

where α (*r*) and α (1) are the tissue attenuation factors for the source-to-detector distances of *r* and 1.0 cm, respectively. Similarly *f* (*r*) and *f* (1) are the exposure-to-dose conversion factors for the distances of *r* and 1.0 cm, respectively, from the source. The values of *f*-factor taken were 0.960, 0.930, 0.930 and 0.930 at distances of 1.0, 5.0, 10.0 and 20.0 cm, respectively.[13]

#### By depth-dose measurement

The measurements in water by 0.1-cc chamber were performed at various distances between 0.8 and 20.0 cm from the source. The measured quantities are affected by the nonuniformity in dose gradient due to chamber size, displacement factor and beam quality. We have used the principle of dose determination near the brachytherapy source for the measurement using the thimble chamber proposed by Tolli *et al*.[29,30] The formula for dose calculation is given by

.......(3), $$D_{W\;}(p_{\mathrm{center}})\;=\;N_{\mathrm{DW}}\;M_{u}\;P_{d}\;K_{n\;}N_{Q}$$

where *N_DW_* is the calibration factor of absorbed dose to water for Co-60 beam. M*_u_* is the measured reading corrected for temperature and pressure. The displacement correction factor used for 0.1-cc chamber was 0.991, which was calculated using the formula from the publication by Tolli *et al*.[30] *N_Q_* is the beam quality correction factor. The displacement correction factor and the beam quality correction factor are constant for all measuring distances. The values of nonuniformity correction factors (*N_n_*) used were 1.107, 1.033, 1.006 and 1.0044 for the source-to-chamber distances of 1.0, 2.0, 5.0 and 8.0 cm, respectively.[27]

The procedure described above was used to determine the dose at various distances (*r*) between 0.8 and 20 cm in water along the transverse axis (θ_*0*_ = 90°) from the GM Plus source. It is well known that the positioning error and nonuniformity correction have major contribution in the uncertainty in dose measurement using the ionization chamber near the brachy source. The effect of these factors decreases rapidly with increase in distance. The nonuniformity correction factor for 0.1-cc chamber at a distance of 5.0 cm from the source is 1.006. The uncertainty in the measured dose due to positioning error is minimized by taking the average dose from measurements performed for at least seven occasions. The uncertainty in the measured dose at 5.0 cm distance can be ignored after applying the nonuniformity correction factor. A number of publications on dosimetry of different ^192^Ir HDR brachytherapy sources have shown that the value of radial dose function at 5.0 cm distance is in the order of 1.000 (±2%).[6,7,9,10] Thus, in addition to the dose at 1.0 cm for the normalization to derive the radial dose function as recommended by TG-43, we have taken the dose at 5.0 cm distance for the normalization in our study. Two different radial dose functions g _L_(*r*) were derived from measured dose along the transverse axis by using the following formulas:

.......(4) $${\left[g_{L}\;\left(r\right)\right]}_{N\;=\;1\;\mathrm{cm}\;}=\;D\left(r,\theta_{0}\right)\;G\left(1,\;\theta_{0}\right)/D\left(1,\;\theta_{0}\right)G\left(r,\theta_{0}\right)$$

.......(5), $${\left[g_{L}\;\left(r\right)\right]}_{N\;=\;5\;\mathrm{cm}\;}=\;D\left(r,\theta_{0}\right)\;G\left(5,\;\theta_{0}\right)/D\left(5,\;\theta_{0}\right)G\left(r,\theta_{0}\right)$$

where *D*(1, θ*_0_*) and *D*(5, θ*_0_*) are the dose rates at 1.0 and 5.0 cm, respectively, along the transverse axis of the source. Similarly *G*(1, θ*_0_*) and *G*(5, θ*_0_*) are the geometry factors at distances of 1.0 and 5.0 cm, respectively, along the transverse axis of the source. The geometry factors were calculated using the formula given by AAPM TG-43.

### Anisotropy function

Special features in GammaMed Plus brachytherapy unit were the positioning accuracy of source within 0.01 cm and the movement with step size of 0.1 cm. This technique was helpful in positioning the source at well-defined points. In our measurements, due to this reason, the chamber was fixed at a particular position taken as origin, and applicator was moved manually at different positions along the transverse axis. The anisotropy functions were measured for the radial distance of 5.0 cm (*r* = 5.0) with polar angles q varying from 10° to 170°. The polar coordinates (*r*, θ) were converted into Cartesian coordinates (*X, Y*). For example, the Cartesian coordinates (*X, Y*) for the polar coordinates (5, 90°), (5, 140°) and (5, 40°) are given by (5, 0), (3.21, 3.83) and (3.21, –3.83), respectively. The *X*-coordinate represents the distance between the chamber and the source applicator. The *Y*-coordinate represents the off-axis distance of source from the line passing through the center of the chamber. Let us consider the measurement setup for the anisotropy factor of *F*(5, 40°). The chamber-to-source applicator distance was taken as 3.2 cm (*X* = 3.2), and the dwell position of the source was chosen in such a way that the off-axis distance was set at –3.8 cm, i.e., *Y* = –3.8. Similar procedures were followed for the measurement at other points. The formula used for the calculation of anisotropy factor is

.......(6), $$F\left(5,\theta \right)=\;D\left(5,\theta \right)\;G\left(5,\theta_{0}\right)/D\left(5,\theta_{0}\right)G\left(5,\theta \right)$$

where *D*(5, θ_0_) and *D*(5, θ) are the dose rates at 5.0 cm along the transverse axis (θ_*0*_) and at angles θ relative to longitudinal axis of the source respectively. Similarly *G*(5, θ_*0*_) and *G*(5, θ) are the geometry factors at 5.0 cm along the transverse axis (θ_*0*_) and at angles θ relative to longitudinal axis of the source respectively.

## Results and Discussion

In our study, we have used MC simulation code for the calculation of some selected dosimetric data to compare with our experimental data using 0.1-cc chamber and other published data. Ballester *et al*.[9] and Taylor *et al*.[10] have done the complete dosimetry of the GammaMed Plus ^192^Ir HDR brachytherapy source using GEANT and Brachydose MC simulation code, respectively. The “Brachydose” is the new user code of EGSnrc code, which has a similar calculation process as DOSRZnrc user code but differs in geometric modeling. It is well known that dosimetric data derived by Meisberger *et al*.[12] are still taken as reference data and perhaps used in some old treatment planning systems. Therefore, as well, we performed experimental study using 0.1-cc ionization chamber to derive tissue attenuation factor, in addition to other data such as radial dose function, and anisotropy function for radial distance of 5.0 cm.

The positioning error was the major source of uncertainty in our measurement, which follows the inverse square law and increases with decrease in the source-to-chamber distance. An accuracy of positioning of canter of the chamber and source applicatorwas best known to be ±0.02 and ±0.01 cm, respectively. Outer and inner diameters of the stainless steel applicator were 1.65 and 1.35 cm, respectively. The overall uncertainty in the measurement of source-to-chamber distance could increase up to ±0.052 cm, including the lateral movement of source inside the applicator. Thus the uncertainty in the measured quantity due to positioning error may be 10%, 5% and 1% at distance of 1.0, 2.0 and 5.0 cm, respectively, from the source. In our experimental setup, the chamber was fixed at one position whereas the source applicator was moved to measuring distance. Reproducibility (*n* = 5) of our measurements by repositioning of the source applicator was found to be within 3%, 1% and 0.1% at measurement distances of 1.0, 5.0 and 10.0 cm, respectively. The large uncertainties in the measured readings for small distances were minimized by the random measurements with a large sample size. The sample size used in this measurement varied from 7 to 12 depending upon the source-to-chamber distance. The standard deviations in radial dose function at various distances were found to be within 2.3% and 2%-5% when derived by using Eq. 5 and 4, respectively. This means the uncertainty in radial dose function is higher when it is derived by normalization with dose measured at 1.0 cm distance than with the dose measured at 5.0 cm distance from the source.

**Figure 2:**
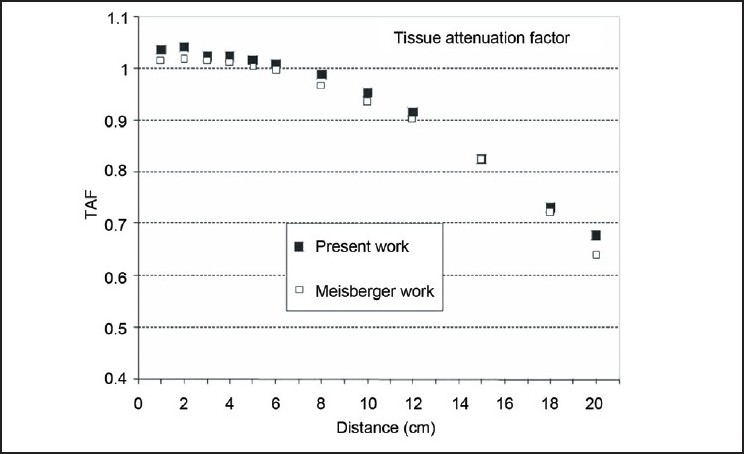
Tissue attenuation factor, comparison of our result with study by Meisberger *et al*

The result of measured tissue attenuation factor vs. distance in our study is plotted and compared with the result from the study by Meisberger *et al*.[12] in Figure 2. The measurement setups used in both the studies were similar. The dimension of the chamber used by Meisberger *et al*.[12] was 1.25 cm length and inner and outer diameters of 0.5 and 0.8 cm, respectively. Our measured data were in good agreement with data by Meisberger *et al*. The maximum difference between these two data was found to be 3.8% at a distance of 20 cm. The data for distances more than 10.0 cm presented for the study by Meisberger *et al*. are calculated from radial dose function. We assumed that the nonuniform photon fluence inside the chamber in water medium does not differ significantly from that in air medium.

The dose rate constant calculated in our study using EGSnrc is shown and compared with results from study by Taylor[10] and Ballester *et al*.[9] for GM Plus, and with results from study by Williamson[6] for Microselectron source in Table 1. The results of dose rate constant are in very good agreement. The dose rate constant of GM Plus source using GEANT code shows a slightly higher value relative to EGSnrc studies. The statistical uncertainties for GM Plus source for all the studies were equal at 0.3%. The ionization chamber has not been used for the measurement of dose rate constant due to higher positioning uncertainty at 1.0 cm distance from the source.

The results of radial dose functions calculated by MC simulation and experimental dosimetry using 0.1-cc chamber are shown in Table 2 and compared with data from study by (i) Taylor[10] and Ballester *et al*.[9] for GM Plus source (ii) Williamson[6] for Microselectron source and (iv) Karaiskos *et al*.[7] and Meigooni[3] for Vari source. Most of the investigators have restricted their measurement to 10.0 cm distance from the source. The published data of radial dose function by Ballester *et al*. using GEANT code[9] and Taylor *et al*. using Brachydose[10] shows very good agreements for all distances taken in the calculation. The results of radial dose function in our study using EGSnrc code show an agreement of within ±2% with the data derived by Taylor *et al*.[10] This ±2% deviation in radial dose function might be due to the differences in voxels size and the number of histories used in the simulation.[31] The results of radial dose function derived by three different methods using 0.1-cc chamber show an agreement within ±3% up to a radial distance of 12.0 cm; after that, for the larger distances it increases up to ±5%. The radial dose functions derived from tissue attenuation factor and depth-dose measurement with normalization at 5.0 cm show better agreement with the data derived by Taylor *et al*.[10] rather than the radial dose function with normalization at 1.0 cm.

**Table 1:** Results of dose rate constant (cGy/h/U) in our study and comparison with other published data

	*Dose rate constant (Λ)*			
*Source*	*Our study*	*Ballester et al.[9]*	*Taylor and Rogers[10]*	*Williamson and Li[6]*
GammaMed	1.115 ±	1.118 ±	1.115 ±	——
Plus	0.003	0.003	0.003	
MicroSelectron	——	——	——	1.115 ± .005

**Table 2:** Results of radial dose function, gL(*r*), in our study and comparison with other published data

Radial dose function gL(*r*)									
*Dist. (cm)*	*Present study (GM Plus Source)*				*Taylor et al.[10]*	*Ballester et al.[9]*	*Williamson et al.[6]*	*Karaiskos et al.[7]*	*Meigooni et al.[3]*
**	*0.1 cc chamber*			*DOSRZ EGSnrc*	*GM Plus*	*GM Plus*	*Micro Sel*.	*Vari*	*Vari*
**	*From TAF*	*[Norm]_1_*	*[Norm]_5_*	*MC Sim*.	*Brachy dose*	*GEANT*	*MCNP*	*MC Sim*	*TLD*
1.0	1.011	1.000	1.029	1.000	1.000	1.000	1.000	1.000	1.000
2.0	0.997	0.985	1.013	1.006	1.004	1.005	1.003	1.010	1.007
3.0	0.989	0.979	1.008	1.024	1.005	1.006	1.002	1.000	1.003
4.0	0.981	0.972	1.002	1.012	1.003	1.006	0.997	······	1.021
5.0	0.982	0.972	1.000	1.009	0.998	1.001	0.987	0.990	0.987
6.0	0.972	0.961	0.989	0.973	0.992	0.994	0.973	0.980	0.966
8.0	0.942	0.933	0.961	0.972	0.969	0.971	0.933	0.940	0.933
10.0	0.906	0.901	0.925	0.922	0.938	0.938	0.871	0.880	0.872
12.0	0.856	0.851	0.873	0.896	0.897	······	0.795	0.800	·····
14.0	····	········	········	·········	0.850	······	0.682	·····	······
15.0	0.771	0.771	0.790	0.829	0.826	······	······	0.610	······
18.0	0.700	0.702	0.707	0.732	0.740	······	·····	·····	······
20.0	0.632	0.637	0.645	0.673	0.683	····	·····	······	·····

The uncertainty in the positioning of the chamber at 1.0 cm could be a major cause of disagreement of the data as the measured value at 1.0 cm has been used for normalization.Since the radial dose function derived from depth-dose function with normalization at 5 cm shows agreement within acceptable limit, the 5 cm distance can be taken as reference distance for the dose measurement using ionization chamber where the positioning uncertainty and effect of nonuniformity correction are minimum. The tissue attenuation factor is independent of position error and nonuniformity correction factor; however, the radial dose function at larger distances (15-20 cm) shows larger deviations in our study. This may be due to the values of exposure-to-dose conversion factor used in calculation.

We observed large deviations in the comparison of radial dose function of GM Plus source with the data from different studies for various other sources. The differences of about 4% to 8% were observed for some distances. This discrepancy over the data of the different sources is due to the dosimetry systems used and the design and construction of the source.

**Figure 3:**
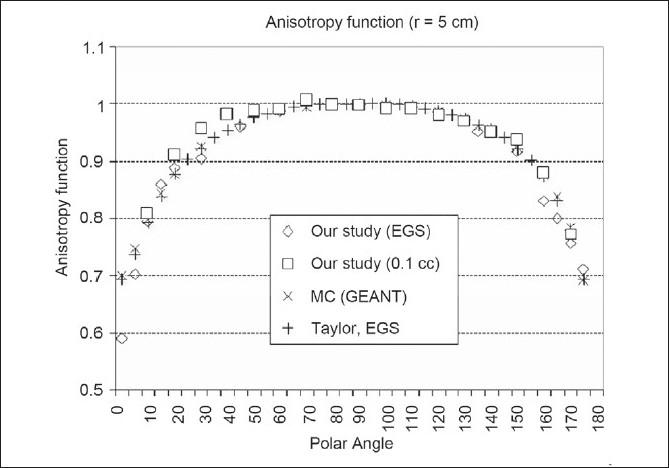
Comparison of anisotropy functions measured for r = 5 cm

The result of anisotropy function *F*(*r*, θ) for radial distances of 1.0, 2.0, 3.0, 5.0 and 10.0 cm with polar angle varying from 0° to 175° for GammaMed Plus source from our Monte Carlo simulation study is shown in Table 3. In comparison of anisotropy function derived from simulation in our study relative to data from the study by Taylor *et al*.,[10] we observed that anisotropy values for radial distance of 5.0 cm were in agreement of about 4% at all polar angles except at 0°. The values were in good agreement at all angles for radial distance of 10.0 cm; however, larger deviations, of about 8%, were observed in values at smaller angles (0°-30°) for radial distance of 1.0 cm. The theoretically and experimentally derived results of anisotropy function for radial distance of 5.0 cm in our study are compared with data derived by Taylor *et al*.,[10] Ballester *et al*.[9] for GammaMed Plus HDR source. The plots of anisotropy function for radial distance of 5.0 cm vs. polar angle (10°-170°) are shown in Figure 3. Our measured values using 0.1-cc chamber show very good agreement, viz., within 3%, at all angles relative to data from the simulation study by Taylor *et al*.[10] and Ballester *et al*.[9] However, deviations of about 1% to 5% were observed in measured values relative to our simulation study.It is to be noted that some angles taken for anisotropy function measurements were not included in our simulation study.The results of anisotropy function from the study by Mishra *et al*.[15] using ion chamber of 0.147 cc volume have shown agreement of about 4% relative to other studies using ion chamber and TLD capsules and MC simulation for microselectron source.

**Table 3:** Results of anisotropy function, *F(r*, θ), for GammaMed Plus source from our MC simulation study

*Anisotropy function F(r,* θ) *for r = 5 cm*																
*Radial distance (cm)*	*Polar angle*															
**	*0°*	*5°*	*10°*	*15°*	*20°*	*30°*	*45°*	*60°*	*90°*	*120°*	*135°*	*150°*	*160°*	*165°*	*170°*	*175°*
1.0	0.674	0.706	0.790	0.859	0.895	0.972	0.972	0.990	1.000	0.978	0.953	0.886	0.820	0.764	0.701	0.627
2.0	0.590	0.629	0.732	0.801	0.835	0.890	0.935	0.957	1.000	0.988	0.965	0.939	0.841	0.754	0.667	0.610
3.0	0.566	0.672	0.755	0.809	0.855	0.928	0.976	1.020	1.000	0.979	0.970	0.925	0.867	0.830	0.707	0.619
5.0	0.592	0.704	0.796	0.861	0.890	0.905	0.961	0.988	1.000	0.987	0.953	0.919	0.832	0.800	0.757	0.713
10.0	0.800	0.825	0.860	0.892	0.925	0.931	0.962	0.995	1.000	0.972	0.957	0.919	0.881	0.849	0.800	0.740

## Conclusions

The ionization chamber with 0.1 cc volume is found to be a suitable dosimetry system for the physical verification of certain dosimetric parameters if proper dosimetric procedure is followed and necessary correction factors are applied. The radial dose function derived from depth-dose measurement and normalized with dose at 5.0 cm distance shows good agreement with simulation-based data. This means the dose at a depth of 5.0 cm can be taken as an alternative for the normalization to derive the radial dose function in the experimental dosimetry of ^192^Ir high-dose rate sources. This may be helpful in minimizing the large uncertainty in the dosimetric data due to the positioning error. The radial dose function derived in our study can be used for treatment planning purposes.
